# Virologic Failures on Initial Boosted-PI Regimen Infrequently Possess Low-Level Variants with Major PI Resistance Mutations by Ultra-Deep Sequencing

**DOI:** 10.1371/journal.pone.0030118

**Published:** 2012-02-15

**Authors:** Max Lataillade, Jennifer Chiarella, Rong Yang, Michelle DeGrosky, Jonathan Uy, Daniel Seekins, Birgitte Simen, Elizabeth St. John, Elizabeth Moreno, Michael Kozal

**Affiliations:** 1 Research and Development, Bristol-Myers Squibb, Wallingford, Connecticut, United States of America; 2 Yale University School of Medicine and Veterans Affairs Health Care Systems, New Haven, Connecticut, United States of America; 3 Research and Development, Bristol-Myers Squibb, Plainsboro, New Jersey, United States of America; 4 454 Life Sciences-Roche Co., Branford, Connecticut, United States of America; Rush University, United States of America

## Abstract

**Background:**

It is unknown whether HIV-positive patients experiencing virologic failure (VF) on boosted-PI (PI/r) regimens without drug resistant mutations (DRM) by standard genotyping harbor low-level PI resistant variants. CASTLE compared the efficacy of atazanavir/ritonavir (ATV/r) with lopinavir/ritonavir (LPV/r), each in combination with TVD in ARV-naïve subjects.

**Objective:**

To determine if VF on an initial PI/r-based regimen possess low-level resistant variants that may affect a subsequent PI-containing regimen.

**Methods/Results:**

Patients experiencing VF on a Tenofovir/Emtricitabine+PI/r regimen were evaluated by ultra deep sequencing (UDS) for mutations classified/weighted by Stanford HIVdb. Samples were evaluated for variants to 0.4% levels. 36 VF subjects were evaluated by UDS; 24 had UDS for PI and RT DRMs. Of these 24, 19 (79.2%) had any DRM by UDS. The most common UDS-detected DRM were NRTI in 18 subjects: M184V/I (11), TAMs(7) & K65R(4); PI DRMs were detected in 9 subjects: M46I/V(5), F53L(2), I50V(1), D30N(1), and N88S(1). The remaining 12 subjects, all with VLs<10,000, had protease gene UDS, and 4 had low-level PI DRMs: F53L(2), L76V(1), I54S(1), G73S(1). Overall, 3/36(8.3%) subjects had DRMs identified with Stanford-HIVdb weights >12 for ATV or LPV: N88S (at 0.43% level-mutational load 1,828) in 1 subject on ATV; I50V (0.44%-mutational load 110) and L76V (0.52%-mutational load 20) in 1 subject each, both on LPV. All VF samples remained phenotypically susceptible to the treatment PI/r.

**Conclusion:**

Among persons experiencing VF without PI DRMs with standard genotyping on an initial PI/r regimen, low-level variants possessing major PI DRMs were present in a minority of cases, occurred in isolation, and did not result in phenotypic resistance. NRTI DRMs were detected in a high proportion of subjects. These data suggest that PIs may remain effective in subjects experiencing VF on a PI/r-based regimen when PI DRMs are not detected by standard or UDS genotyping.

## Introduction

HIV treatment guidelines recommend combination antiretroviral (ARV) regimens for treatment naive patients consisting of a nucleoside/nucleotide reverse transcriptase inhibitor (N(t)RTI)-based backbone along with either a non-nucleoside reverse transcriptase inhibitor (NNRTI), an integrase inhibitor (INI) or a ritonavir (RTV)-boosted protease inhibitor (PI) [1]. At the end of 2009, 584,000 HIV positive patients under care were receiving ARVs in the USA [2]. Approximately 40% of patients were initiated on an N(t)RTI+RTV-boosted PI (PI/r) as a first line regimen [2].

The most common drug resistance mutation pattern by standard HIV genotyping in patients experiencing virologic failure on an initial regimen of Tenofovir/Emtricitabine (TDF/FTC) plus a ritonavir boosted protease inhibitor (PI/r) is a solitary reverse transcriptase (RT) M184V mutation or no resistance mutations [3]–[7]. It is not known if HIV patients on a PI/r regimen experiencing virologic failure without evidence of PI resistance mutations by standard genotyping harbor low-level PI-resistant variants that are below the detection levels of standard genotyping methods. Further, if low-level resistant variants exist in these patients, could they affect the use of a subsequent PI/r containing regimen?

The objective of this study was to determine if patients with virologic failure on an initial PI/r-based regimen without resistance by standard genotyping possess low-level drug resistant variants that could affect the use of a subsequent PI/r containing regimen. To investigate this question, ultra deep sequencing was performed on the virologic failure specimens from the CASTLE study [5]–[7]. CASTLE compared the efficacy of atazanavir/ritonavir (ATV/r) with lopinavir/ritonavir (LPV/r), each in combination with TDF/FTC in ARV-naïve patients through 96 weeks of treatment [5]–[7].

## Methods

**Figure 1:**
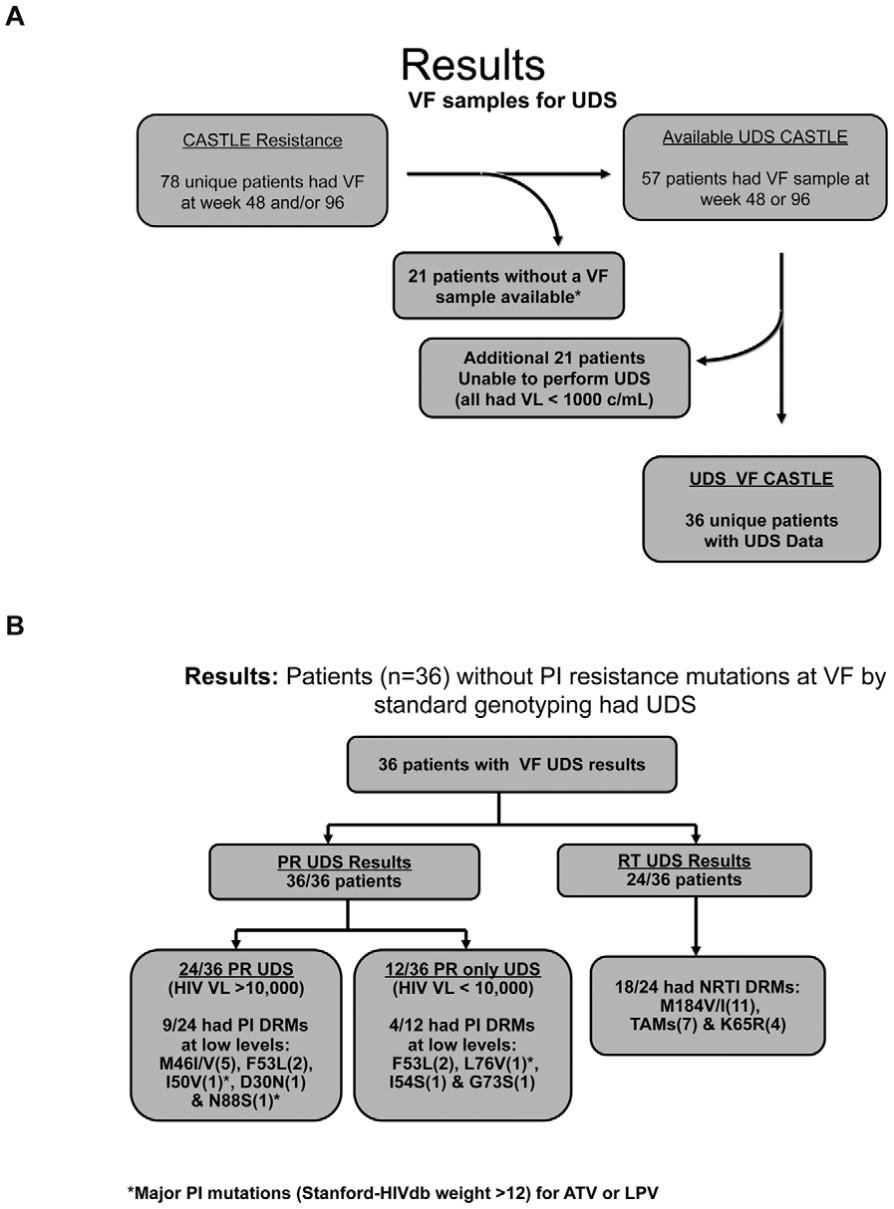
Description and UDS results for virologic failure samples. **a:** Virologic failure samples for UDS. UDS: Ultra Deep Sequencing; VF: Virologic Failure. 78 subjects had virologic failure at week 48 and/or 96. *21 samples were either exhausted or could not be located. 57 patients with virologic failure without PI resistance had samples for UDS. 21 patients failed with HIV RNA<1,000 copies and UDS could not be performed. 36 unique patients had UDS data. **b:** UDS results for 36 Virologic Failures. VF: Virologic Failure; PR: Protease; RT: Reverse Transcriptase. UDS: Ultra Deep Sequencing; DRMs: Drug Resistance Mutations. 36 patients without PI resistance mutations at VF by standard genotype were evaluated by UDS. 36 patients with VF had PR evaluated by UDS. 24/36 samples had HIV VL>10,000 c/ml. 9/24 had PI DRMs at low levels. 12/36 samples had HIV VL<10,000 c/ml. 4/12 had PI DRMs at low levels. Only 3/36 patients with VF had PI mutations with HIVdb weight >12 for ATV or LPV. 24/36 patients with HIV VL>10,000 c/ml had RT evaluated by UDS. 18/24 samples had NRTI DRMs.

All available Week 48 and 96 CASTLE virologic failure specimens from patients without PI resistance mutations by standard genotype were evaluated by ultra deep sequencing (454-Life Sciences/Roche, Branford, CT) for N(t)RTI+PI/r mutations (see Figure 1a) and classified by Stanford HIVdb algorithm [8]. Virologic failure specimens with HIV viral loads >1,000 copies/mL allowed for ultra deep sequencing to be attempted. Ultra deep sequencing was performed on both the protease (PR) and RT genes for virologic failure specimens with HIV viral load >10,000 c/mL. Virologic failure specimens with HIV viral loads <10,000 c/mL had ultra deep sequencing performed only for the PR gene. All drug resistance mutations and polymorphisms were evaluated by Stanford HIV db algorithm; specific PI mutations with a value >12 for the PI used in the study (ATV and LPV) were considered significant resistance mutations. Ultra deep sequencing was performed as described in previous studies [9]–[10]. Ultra deep sequencing was performed to 0.4% variant detection levels depending upon the sample HIV viral load. Sampling of HIV RNA from a plasma sample follows Poisson distribution and is subject to the stochastic effects of sampling variation [9], [11], [12]. An estimated mutational load (ML) was calculated by percent of variant detected x sample viral load (Roche Amplicor Assay). Historical standard genotypes and phenotypes were available on all CASTLE specimens for baseline and virologic failure samples (Monogram Biosciences, South San Francisco, CA). Susceptibility cut-off used for TDF, FTC, ATV/RTV and LPV/RTV in CASTLE were clinical cut-offs (fold change-FC) from Monogram Biosciences (TDF>1.4, FTC/3TC>3.5, ATV/RTV>5.2, LPV/RTV>9).

All IRB approvals for this study were already obtained under the parent CASTLE study. All subjects had given informed consent. The parent CASTLE study is registered with ClinicalTrials.gov, number NCT00272779.

## Results

Of 78 patients with virologic failure at Weeks 48 and/or 96 from the CASTLE study without PI resistance mutations by standard genotype, 57 were available for ultra deep sequencing (Figure 1a). Of the 57 virologic failure specimens, 36 had a viral load >1,000 copies/mL, which allowed for ultra deep sequencing to be attempted. Ultra deep sequencing was performed on both the PR and RT genes for all 24 (66.7%) specimens with HIV viral load >10,000 c/mL. The remaining 12 (33.3%) virologic failure specimens with HIV viral loads <10,000 c/mL had ultra deep sequencing performed only for the PR gene (Figure 1a). For the 24 samples with ultra deep sequencing for both PR and RT, 5/24 (20.8%) were wild type at all codon positions (no resistance mutations). Nineteen of 24 (79.2%) had any PI, N(t)RTI and/or NNRTI resistance mutations; 18/24 (75%) had N(t)RTI mutations: M184V/I (11), TAMs(7) and K65R (4), and 9/24 (37.5%) had PI mutations: M46I/V(5), F53L(2), I50V(1), D30N(1) & N88S(1) (Figure 1b). Of the 12 specimens that had ultra deep sequencing of only the protease gene, 4/12 (33.3%) had low level PI mutations: F53L(2), L76V(1), I54S(1) and G73S(1) (Figure 1b).

Overall, 3/36 (8.3%) patients had PI mutations identified with Stanford-HIVdb weights >12 for ATV or LPV: 1 patient on ATV with **N88S** (0.43%-mutation load 1,828) and 2 patients on LPV: one with **I50V** (0.44%-mutation load 110) and the remaining patient with **L76V** (0.52%-mutation load 20). The mutations identified by UDS in each specimen and the estimated mutational loads with the corresponding phenotypes are listed in Table S1 and Table S2. All samples with low-level variants with PI mutations remained phenotypically susceptible to PIs (Table S1 and Table S2-Historical phenotypes). Out of 11 patients with M184V/I at virologic failure, 4 with M184V developed phenotypic resistance to FTC/3TC (variant range 95.93% to 100%; phenotypic range 57.54 to 75.63). Of the seven patients with a M184V/I without phenotypic resistance to 3TC/FTC, the variant levels ranged from 0.42% to 31.1%. Four patients had a K65R at virologic failure (variant range 0.52% to 1.28%), and none developed phenotypic resistance to TDF (Table S1).

## Discussion

The results from this study demonstrated that among patients on an initial PI/r based regimen experiencing virologic failure without PI resistance mutations by standard genotyping, low-level variants possessing major PI mutations were present in a minority of cases, occurred in isolation, and did not result in phenotypic PI resistance. Only 3 patients (8.3%) had a PI mutation with a Stanford algorithm weight >12 for the PIs used in the study (ATV or LPV). However, they were at very low levels with low mutational loads. It should be noted that none of the low-level variants with a major PI mutation were at levels ≥1% of the viral population, a level that has been shown to be clinically significant for NNRTIs based regimens [9]. However, the PI mutations identified in these specimens were known to be associated with drug resistance to the corresponding PI. Of note, N(t)RTI resistance mutations were detected in a high proportion (75%) of virologic failure specimens and with greater frequency than PI mutations, which may partly explain the viral breakthrough in these patients. Interestingly, not all patients with M184V/I developed phenotypic resistance to FTC/3TC. Eleven of 24 patients had low or high level M184V/I variants; 7 had M184V/I at ≥1% of the viral population, however only 4 of the 7 developed phenotypic resistance to FTC/3TC (all had M184V variant levels >95%). None of the patients with K65R (all at <2%) developed phenotypic resistance to TDF (See Table S1).

These findings underscore the limitation of stand-alone phenotypic susceptibility results and emphasize the importance of complementary and/or more sensitive genotyping techniques when evaluating for resistance [13]. In addition, these data further suggest that the impact of drug resistance mutations on therapy is multi-factorial and should take into account the proportion of the variant present, the mutational load, and the specific mutation present [10].

As previously reported by our group, low-level variants possessing PI mutations are infrequently identified in treatment-naïve persons and did not significantly affect the efficacy of PI/r based regimens [14], a finding in contrast to low-level NNRTI resistance mutations and NNRTI-based regimens [9]. In this study, we have shown that low-level PI drug resistant variants are also infrequently detected by ultra deep sequencing when patients experience virologic failure on TDF/FTC+PI/r without PI mutations detected by standard genotype.

The results of this study may have implications for first line and subsequent ARV sequencing strategies. According to current guidelines, second line ARV regimens for subjects with resistance to NNRTI or PI/r based regimen should consist of at least 2, preferably 3 new and fully susceptible ARVs [1]. This decision should be based on drug history, genotypic and phenotypic data, and the mechanism of action of the new drugs. Of note, drug potency and viral susceptibility are as important as the number of new drugs prescribed [1]. As shown in this study, patients who failed a PI/r regimen in CASTLE without PI resistance by standard genotype while remaining phenotypically susceptible infrequently had low level variants resistant to the PI/r used or to any other relevant PI/r. This data suggest that boosted PIs may still provide good safety and efficacy and could possibly be re-used in later lines of therapy with a different ARV backbone if no PI mutations are present by ultra deep sequencing and as long as adherence is addressed. However, this hypothesis would need to be formally tested in clinical studies.

Our study had several limitations. Given the requirement for other predetermined study analyses we were only able to recover specimens for 57 of 78 Week 48 and/or 96 CASTLE virologic failures. Further, we were unable to perform ultra deep sequencing on 21 virologic failure specimens that had an HIV viral load ≤1,000 c/mL. Full length reverse transcriptase ultra deep sequencing for samples with HIV VL>10,000 c/mL was limited to 24 samples. Given stochastic effects of RNA sampling for the samples with HIV VL<10,000 c/mL, the reported variants may or may not represent the proportion of variants in plasma. Thus, we are unable to confidently analyze all specimens to low levels which may have lead to an undetected selection bias.

### Conclusion

In conclusion, among patients on an initial PI/r based regimen experiencing virologic failure without PI mutations by standard genotyping, low-level variants possessing major PI mutations by ultra deep sequencing were infrequently identified and if PI mutations were present they were found in isolation and did not result in phenotypic PI resistance. Only 3 patients (8.3%) had a PI mutation with a Stanford algorithm weight >12 for ATV or LPV, however they were at very low levels (<1%) with low mutational loads. NRTI mutations were detected in a high proportion of patients with virologic failure and with greater frequency than PI mutations. These data suggest that PIs may remain effective in patients experiencing virologic failure on a PI/r-based regimen, and may be re-used in subsequent ARV regimens with new optimized background therapy when PI resistance are not detected by standard or ultra deep sequencing. As the HIV population grows older, clinicians will need to conserve agents and use potent and relatively safer ARVs for as long as possible.

These data were presented in part at the 2010 International HIV & Hepatitis Virus Drug Resistance Conference. Dubrovnik, Croatia, Abstract #102.

## Supporting Information

Table S1 **UDS data for RT and PR and phenotype for 24/36 VF subjects.** Ultra deep sequencing, mutational load, and phenotypic susceptibility results for 24 samples from subjects experiencing virologic failure on TDF+FTC+PI/r. # UDS for two virologic failure (VF) timepoints – both VF specimens had a M184V mutation. NRTI DRMs: M184V and K65R are bolded in black. PI DRMs with a Stanford HIVdb weight >5 are bolded in black with an asterix *. Please note that if a sample had different HIV variants with the same mutation they are listed separately e.g. Subject #2 had different HIV variants with a M184V at 3.92% and M184V at 94.94%.
(DOCX)

Table S2 **UDS data for PR only and phenotype for 12/36 VF subjects.** Ultra deep sequencing and mutational load for PIs only, and phenotypic susceptibility results for 12 samples from subjects experiencing virologic failure on TDF+FTC+PI/r. PI DRMs with a Stanford HIVdb weight >5 are bolded.
(DOCX)
