# Supplementary material for: Virologic Failures on Initial Boosted-PI Regimen Infrequently Possess Low-Level Variants with Major PI Resistance Mutations by Ultra-Deep Sequencing
Source: PLoS One. 2012 Feb 15;7(2):e30118. doi: 10.1371/journal.pone.0030118 (PMC3280244; doi:10.1371/journal.pone.0030118)
Supplement: Table S1 — UDS data for RT and PR and phenotype for 24/36 VF subjects. Ultra deep sequencing, mutational load, and phenotypic susceptibility results for 24 samples from subjects experiencing virologic failure on TDF+FTC+PI/r. # UDS for two virologic failure (VF) timepoints – both VF specimens had a M184V mutation. NRTI DRMs: M184V and K65R are bolded in black. PI DRMs with a Stanford HIVdb weight >5 are bolded in black with an asterix *. Please note that if a sample had different HIV variants with the same mutation they are listed separately e.g. Subject #2 had different HIV variants with a M184V at 3.92% and M184V at 94.94%. (DOCX) [file pone.0030118.s001.docx]

| **Table S1. UDS data for RT and PR and phenotype for 24/36 VF subjects** | | | | | | | | | | | | | | | |
| --- | --- | --- | --- | --- | --- | --- | --- | --- | --- | --- | --- | --- | --- | --- | --- |
| **Patient ID** | **Regimen** | **CLADE** | **VL VF** | | **UDS** | | | | | | **NRTI + PI Mutational Load** | **Phenotype** | | | |
|  |  |  |  |  | **NRTI** | | | **NNRTI** | | **PI** |  | **FC ATV** | **FC LPV** | **FC FTC** | **FC TDF** |
| 1 | LPV | B | 7,900 | | K70N(1.18%), **M184V(26.29%),** K219E(54.5%) | | |  | | I13V(0.77%), L33I(0.75%), L33V(99.21%), **M46I(0.72%**)*, I64V(96.2%), I64V(2.77%), I64V(0.66%) | K70N(93), **M184V(2,077),** K219E(4,306), *M46I(57)* | 1.06 | 0.84 | 2.41 | 0.55 |
| 2 | LPV | B | 16,500 | | M41L(0.67%), **M184V(3.92%), M184V(94.94%**) | | | K103E(0.73%) | | I13V(1.89%), M36I(0.75%), D60E(99.68%), I62V(83.51%), L63P(99.9%), I64V(16.91%), V77I(1.69%), V82I(2.26%), V82I(97.23%) | M41L(111**), M184V(647), M184V(15,665)** | 1.04 | 0.81 | **>57.54** | 0.66 |
| 3 | LPV | B | 16,800 | | **M184I(0.56%**) | | | K103E(0.51%) | | I13V(1.55%), M36I(17.12%), L63P(100%), I64M(0.4%), A71V(99.27%), V77I(99.77%), I93L(99.74%) | **M184I(94)** | 1.11 | 1.17 | 1.00 | 0.81 |
| 4 | LPV | B | 21,400 | | NONE | | | L100V(8.41%) | | I13V(1.52%), M36I(0.67%), D60E(99.82%), V77I(99.73%) | NONE | 1.2 | 1.18 | 1.23 | 0.97 |
| 5 | LPV | B | 25,100 | | NONE | | | L100V(9.34%) | | L10I(1.43%), I13V(0.44%), **I50V(0.44%)***, I62V(72.2%), I62V(27.73%), L63P(100%), V77I(20.21%), I93L(99.3%), I93L(0.64%) | *I50V(110)* | 0.98 | 0.54 | 1.24 | 0.87 |
| 6 | LPV | B | 35,300 | | A62V(0.52%), T215I(0.47%) | | |  | | I13V(2.94%), M36I(1.24%), **M46I(1.21%)***, D60E(98.53%), I62V(99.3%), L63P(99.76%), A71T(2%), V77I(99.72%), I93L(99.44%) | *M46I(427)* | 0.62 | 0.55 | 0.94 | 0.96 |
| 7 | LPV | B | 65,500 | | NONE | | |  | | I13V(1.18%), M36I(0.86%), I62V(0.6%), I62V(99.33%), L63P(98.83%), I64L(86.97%), I64M(11.53%), V82I(78.66%), I93L( 95.07%) | NONE | 0.62 | 0.55 | 0.87 | 0.82 |
| 8 | LPV | B | 321,000 | | **M184V(31.1%), M184I(0.57%**) | | | G190E(0.46%) | | I13V(1.94%), M36I(37.74%), **M46V(0.73%)***, L63P(0.41%), I64M(0.41%), I93L(98.89%) | **M184V(99,831), M184I(1,830),** **M46V(2,343)*** | 0.48 | 0.42 | 1.23 | 0.69 |
| 9 | LPV | BF | 61,000 | | V75A(1.29%) | | | K103E(2.45%) | | I13V(100%), M36I(2.24%), **M46I(1.12%)***, I64V(99.53%), V77I(95.69%) | V75A(787), **M46I(683)*** | 0.75 | 0.77 | 1.02 | 0.94 |
| 10 | LPV | C | 13,600 | | **K65R(0.65%), M184V(95.93%)** | | | V106A(0.78%) | | I13V(0.95%), G16E(15.88%), H69K(94.11%), A71T(95.03%), V77I(99.79%), I93L(99.96%) | **K65R(88), M184V(13,046)** | 0.48 | 0.39 | **>62.09** | 0.52 |
| 11 | LPV | C | 97,400 | | **K65R (0.76%)**, D67G (0.42%) | | |  | | I13V(5.11%), K20R(98.66%), M36I ( 99.65%), I62V(0.46%), L63P(34.71%), H69K(94.74%), V82I(55.26%), I93L(99.6%) | **K65R(740),** D67G(409) | 2.74 | 1.49 | 0.9 | 1.06 |
| 12 | LPV | C | 159,000 | | D67G(0.43%), T69A(1.03%), T69S(1.84%), V75A(0.43%), **M184I(1.03%), M184V(0.42%)**, T215I(1.33%) | | |  | | I13V(2.41%), G16E(0.53%), M36I(31.55%), M36L(67.68%), D60E(98.23%), I62V(19.5%), L63P(96.09%), L63P(3.58%), H69K(95.85%), I93L(99.47%) | D67G(683), T69A(1,637), T69S(2,926), V75A(684), **M184I(1,638), M184V(668)**, T215I(2,115) | 1.33 | 0.89 | 0.93 | 0.86 |
| 13# | LPV | C | 555,000/ 75,500 | | **K65R(0.71%)**, **M184V(99.77%)/M184V(98.35%**) | | | K101E(1.07%), K101E(0.71%) | | L10I(97.98%), L10I(0.69%), L10V(0.4%), I13V(96.72%), M36I(99.42%), **F53L(0.69%)***, I64V(0.46%), H69K(98.22%), A71T(0.46%), I93L(92.11%) | **K65R(536), M184V(75,326) / M184V(545,843), F53L(521)*** | 0.63 | 0.55 | **>62.09** | 0.53 |
| 14 | ATV | AE | 425,000 | | K70N(0.57%), F77L(1%) | | |  | | **N88S(0.43%**)* | K70N(0.57%), F77L(4250), **N88S(1828)*** | 0.84 | 0.74 | 0.64 | 0.52 |
| 15 | ATV | B | 10,500 | | T215I (0.66%) | | |  | | I13V(98.23%), M36I(3.81%), I62V(0.78%), I64V(99.48%), V82I(1.91%) | T215I(69) | 0.74 | 0.56 | 0.75 | 0.93 |
| 16 | ATV | B | 11,800 | | NONE | | |  | | L10R(0.45%), I13V(2.86%), K20R(98.76%), M36I(97.97%), H69K(94.67%), I93L(98.68%), I93L(0.44%) | NONE | 0.97 | 0.75 | 0.99 | 0,79 |
| 17 | ATV | B | 31,800 | | M41L(96.53%), T215D(99.24%) | | | K103E(0.53%) | | I13V(3.29%), D60E(0.93%), D60E(98.74%), I62V(80.53%), L63P(52.22%), V77I(1.47%) | M41L(30,697), T215D(31,558) | 0.6 | 0.52 | 1.01 | 0.78 |
| 18 | ATV | B | 35,500 | | T69N(1.69%), **M184I(0.62%)** | | | K103E(0.87%), K103R(57.34%), K103S(39.69%), Y181C(6.95%) | | I13V(2.23%), I62V(23.37%), L63P(100%), I64L(1.47%), V77I(64.96%), I93L(99.43%) | T69N(600), **M184I(220)** | 0.82 | 0.79 | 1.02 | 0.89 |
| 19 | ATV | B | 542,000 | | T69A(5.04%), **M184V(0.5%)**, K219E(0.42%) | | | P225H(1%) | | I13V(0.61%), **D30N(0.67%)***, M36I(0.91%), L63P(14.33%), I64V(0.54%), A71T(25.81%), V77I(24.71%) | T69A(27,317), **M184V(2,710)**, K219E(2,276)**, D30N(3,631)*** | 0.78 | 0.88 | 0.96 | 0.97 |
| 20 | ATV | BF | 9,890 | | **M184V(100%**) | | |  | | I13V(0.65%), K20R(5.07%), M36I(96.83%), L63P(97.91%), L63P(2.09%), V82I(1.6%) | **M184V(9,890)** | 1.11 | 1.34 | **>75.63** | 0.48 |
| 21 | ATV | BF | 28,000 | | NONE | | |  | | I13V(0.46%), G16E(1.29%), M36I(43.31%), M36V(55.7%) | NONE | 1.34 | 1.53 | 0.97 | 0.79 |
| 22 | ATV | C | 12,300 | | **M184V(0.43%**) | | | L100V(5.01%) | | I13V(1.4%), M36I(98.31%), **M46V(0.77%)*, F53L(0.51%)***, L63P(95.77%), H69K(99.49%), V77I(0.6%), I93L(99.94%) | **M184V(53), M46V(95)*, F53L(63)*** | 0.69 | 0.54 | 0.96 | 1.00 |
| 23 | ATV | C | 92,000 | | NONE | | |  | | I13V(1.85%), M36I(98.53%), M36I(0.49%), L63P(99.51%), H69K(95.95%), I93L(98.78%), I93L(0.84%) | NONE | 0.78 | 0.77 | 0.75 | 0.97 |
| 24 | ATV | C | 180,000 | | **K65R(1.28%), K65R(0.52%)** | | |  | | I13V(0.57%), G16E(95.03%), H69K(92.69%), H69K(6.43%), V77I(99.87%), I93L(99.54), I93L(0.46%) | **K65R(2,304), K65R(936)** | 1.65 | 1.37 | 1.19 | 1.09 |
|  | | | | | | | | | | | | | | | |
|  | | | |  | |  |  | |  |  |  |  |  |  |  |
|  | | | |  | |  |  | |  |  |  |  |  |  |  |
|  | | | |  | |  |  |  |  |  |  |  |  |  |  |
