# Supplementary material for: Virologic Failures on Initial Boosted-PI Regimen Infrequently Possess Low-Level Variants with Major PI Resistance Mutations by Ultra-Deep Sequencing
Source: PLoS One. 2012 Feb 15;7(2):e30118. doi: 10.1371/journal.pone.0030118 (PMC3280244; doi:10.1371/journal.pone.0030118)
Supplement: Table S2 — UDS data for PR only and phenotype for 12/36 VF subjects. Ultra deep sequencing and mutational load for PIs only, and phenotypic susceptibility results for 12 samples from subjects experiencing virologic failure on TDF+FTC+PI/r. PI DRMs with a Stanford HIVdb weight >5 are bolded. (DOCX) [file pone.0030118.s002.docx]

| **Table S2. UDS data for PR and phenotype for 12/36 VF subjects** | | | | | | | | | |
| --- | --- | --- | --- | --- | --- | --- | --- | --- | --- |
| **Patient ID** | **Regimen** | **CLADE** | **VL VF** | **UDS PI Mutations only** | **PI Mutational load** | **FC ATV** | **FC LPV** | **FC FTC** | **FC TDF** |
| 25 | LPV | B | 1,860 | I13V(0.5%), G16E(43.77%), M36I(53.58%), I62V(46.23%), I64V(2.29%), A71T(8.06%), V77I(34.75%) | NONE | 0.92 | 0.79 | 1.15 | 0.87 |
| 26 | LPV | B | 3,440 | G16E(95.29%), I62V(99.88%), V77I(99.87%) | NONE | 0.61 | 0.78 | 1.12 | 0.76 |
| 27 | LPV | B | 3,780 | I13V(0.6%), M36I(1.02%), **F53L(0.43%)**, I62V(90.21%), L63P(99.01%), I64V(1.06%), **L76V(0.52%),** V77I(99.63%), I93L(98.21%) | **F53L(16), L76V(20)** | 0.72 | 0.72 | 1.09 | 0.76 |
| 28 | LPV | B | 4,070 | L33V(1.95%), M36I(4.33%), **I54S(0.45%)**, I62V(1.84%), L63P(98.24%), A71T(0.55%), V77I(1.15%), I93L(0.48%) | **I54S(18)** | 0.48 | 0.5 | 0.8 | 0.93 |
| 29 | LPV | BF | 5,370 | I13V( 0.63%),G16E(1.36%),M36I(97.85%), M36L(1.3%), D60E(1.06%), L63P(67.37%), I64V(6.38%), H69L(0.55%), V77I(98.47%), I93L(98.99%) | NONE | 2.09 | 1.48 | 0.65 | 0.76 |
| 30 | LPV | BF | 5,860 | I13V(0.42%), M36I(99.45%), I64M(2.99%), A71T(5.97%) | NONE | 1.75 | 1.35 | 0.99 | 0.86 |
| 31 | ATV | B | 8,380 | L10V(99.24%), I13V(0.54%), M36I(28.32%), **F53L(1.53%)**, A71V(99.76%), V77I(99.78%), I93L(99.91%) | **F53L(128)** | 2.14 | 1.57 | 1.1 | 0.89 |
| 32 | ATV | BF | 1,690 | I13V(0.94%), K20R(2.7%), M36I(98.91%), I62V(6.22%) | NONE | 0.94 | 1.01 | **>46.73** | 0.45 |
| 33 | ATV | C | 2,510 | I13V(1.43%), M36I(99.46%), H69K(98.66%), I93L(99.1%) | NONE | 0.83 | 0.53 | 0.62 | 0.51 |
| 34 | ATV | C | 2,880 | I13V(0.69%), K20R(95.92%), M36I(98.72%), H69K(97.81%), V82I(5.59%), I93L(99.93%) | NONE | 1.32 | 0.97 | 0.92 | 0.66 |
| 35 | ATV | C | 4,520 | L10R(1.11%), I13V(2.07%), G16E(96.97%), L33V(0.58%), M36I(47.63%), D60E(2.24%), D60E(97.6%), I62V( 13.24%), H69K(0.57%), H69K(70.14%), I93L(99.42%) | NONE | 0.88 | 0.55 | 1.04 | 0.86 |
| 36 | ATV | C | 6,900 | I13V(1.49%), K20R(96.99%), M36I(98.67%), D60E(7.87%), H69K(98.1%), **G73S(5.4%)**, I93L(99.68%) | **G73S(373)** | 0.91 | 0.5 | 0.87 | 0.74 |
